# Medium throughput bisulfite sequencing for accurate detection of 5-methylcytosine and 5-hydroxymethylcytosine

**DOI:** 10.1186/s12864-017-3489-9

**Published:** 2017-01-18

**Authors:** Gary G. Chen, Jeffrey A. Gross, Pierre-Eric Lutz, Kathryn Vaillancourt, Gilles Maussion, Alexandre Bramoulle, Jean-François Théroux, Elena S. Gardini, Ulrike Ehlert, Geneviève Bourret, Aurélie Masurel, Pierre Lepage, Naguib Mechawar, Gustavo Turecki, Carl Ernst

**Affiliations:** 1 Department of Psychiatry, McGill Group for Suicide Studies, Douglas Mental Health University Institute, McGill University, Montreal, 6875 boul. LaSalle, Verdun, QC H4H 1R3 Canada; 2 University of Zurich, Clinical Psychology and Psychotherapy, Zurich, Switzerland; 3 Genome Quebec Innovation Centre, McGill University, Montreal, QC Canada

**Keywords:** Targeted bisulfite sequencing, 5-methylcytosine, 5-hydroxymethylcytosine, Next-generation sequencing, Customized MiSeq sequencing, Multiplexed PCR-directed BS-amplicons, Multiplexed barcoding of target amplicon libraries

## Abstract

**Background:**

Epigenetic modifications of DNA, such as 5-methylcytosine and 5-hydroxymethycytosine, play important roles in development and disease. Here, we present a cost-effective and versatile methodology for the analysis of DNA methylation in targeted genomic regions, which comprises multiplexed, PCR-based preparation of bisulfite DNA libraries followed by customized MiSeq sequencing.

**Results:**

Using bisulfite and oxidative bisulfite conversion of DNA, we have performed multiplexed targeted sequencing to analyse several kilobases of genomic DNA in up to 478 samples, and achieved high coverage data of 5-methylcytosine and 5-hydroxymethycytosine at single-base resolution. Our results demonstrate the ability of this methodology to detect all levels of cytosine modifications at greater than 100× coverage in large sample sets at low cost compared to other targeted methods.

**Conclusions:**

This approach can be applied to multiple settings, from candidate gene to clinical studies, and is especially useful for validation of differentially methylated or hydroxymethylated regions following whole-genome analyses.

**Electronic supplementary material:**

The online version of this article (doi:10.1186/s12864-017-3489-9) contains supplementary material, which is available to authorized users.

## Background

Epigenetic modifications have been associated with many aspects of mammalian development and physiology [1, 2]. In particular, the most widely studied epigenetic marks have been DNA cytosine modifications, namely 5-methylcytosine (5mC) and 5-hydroxymethylcytosine (5hmC). Currently, achieving high-resolution, genome-wide detection of cytosine modifications in sufficient sample sizes to make claims about disease association at affordable cost remains a challenge. In mapping DNA modifications, the most commonly used technologies include whole genome bisulfite sequencing (WGBS), which maps cytosine methylation across the entire genome at single base resolution. Although this method, theoretically, covers every CpG (~30 million) in the human genome, getting adequate coverage on each individual CpG site is a costly and formidable task. Another genome-wide DNA modification methodology, reduced representation bisulfite sequencing (RRBS) [3, 4], utilizes methylation-insensitive restriction enzyme digestion and size selection to reduce the amount of genetic material being sequenced. Unlike WGBS, RRBS tends to extensively focus on CpG-rich regions [5, 6]. Finally, array-based technologies are also popular but these are limited to genomic regions covered by the pre-selected probes. Still, whether by WGBS, RRBS, or arrays, genome-wide identification of differentially methylated regions (DMRs) often requires a technical validation and/or replication in additional cohorts, and deep sequencing of specific target regions is often called for.

Target validation strategies primarily employ next-generation sequencing (NGS) of libraries prepared from target regions from bisulfite-converted (BS) genomic DNA. The most frequently used library preparation techniques include, but are not limited to, Illumina’s TruSeq LT and HT or Nextera technologies, which can provide precise and accurate measures of DNA modifications. The TruSeq Custom Amplicon strategy from Illumina has been recently introduced for amplicon sequencing and the interrogation of custom regions of interest. However, this solution relies on the TruSeq Custom Amplicon Library Preparation Kit and the use of their DesignStudio software for designing amplicon primers, which is not only costly but also limited to the use of Illumina’s barcoding and sequencing system. The DesignStudio software generates custom oligonucleotide panels that are intended for the amplification of genomic DNA and are not applicable, at this point, to bisulfite-converted DNA. Therefore, for DNA methylation studies, one needs to use the TruSeq LT methyl adaptor kit from Illumina, which is restricted to 12 single indices. This situation significantly hampers the ability of clinical and research laboratories to carry out differential methylation analyses in large collections of genomic regions and sample sets. In addition, these approaches rely on enzymatic library preparations and remain expensive and cumbersome to non-experienced users.

Here, we present and characterize a robust approach for targeted BS-sequencing, which incorporates target amplification and library generation into three consecutive PCR reactions. Our approach eliminates the need for expensive amplicon library preparation kits, uses freely available web-based software for BS-primer design, and only requires a set of reusable, universal primers for barcoding, library preparation, and customized sequencing. Our methodology is highly affordable and efficient, and has been validated, here, in several tissues over multiple target regions. We show the ability of this approach to simultaneously interrogate multiple loci in 478 samples in a single sequencing run. Our unique single barcoding system can be easily extended to 1536 indices and, therefore, outperforms the low-multiplexing and costly Illumina dual barcoding system. Our method allows users to sequence hundreds of bisulfite or oxidative bisulfite converted DNA regions ranging from 100 bases up to several kilobases in a single and customized MiSeq run. Whether investigating psychiatric phenotypes in peripheral samples, uniparental disomy in developmental phenomena, or tumor biopsies in cancer patients, this methodology will prove valuable as a method to rapidly and cost-effectively evaluate cytosine modifications in large sample cohorts and extended genomic regions.

## Methods

### Subjects

Saliva samples were collected using the Oragene Saliva self-collection Kit (DNA Genotek, cat. # OG-500) and stored at room temperature. DNA was extracted using the prepIT-L2P kit (DNA Genotek, cat. # PT-L2P). Post-mortem human brain tissue from adult male subjects was obtained from biorepositories at the Douglas – Bell Canada Brain Brank. All tissue was dissected at 4 **°**C, snap-frozen in liquid nitrogen, and stored at -80 **°**C following standard procedures. The Quebec Coroner’s office assessed the cause of death for each subject and, subsequently, we obtained information on the subjects’ mental health using psychological autopsies using the Structured Clinical Interviews for DSM-IV Axis 1. In addition, brain tissue samples from all subjects were assessed for absence of pathological processes by a neuropathologist. Genomic DNA was extracted from brain tissue using QIAGEN’s QIAmp DNA Mini Kit (QIAGEN, cat #: 51304). For DNA from saliva and brain, NanoDrop 2000 spectrophotometer and Quant-IT PicoGreen (Thermo Scientific, cat. #: P7589) were used to assess the DNA quality and concentration. Written informed consent was obtained from next-of-kin and the Douglas Institute Research Ethics Board approved this study.

### Cell culture

An induced pluripotent stem cell-derived neural progenitor cells (iPSC-NPC) line was generated from skin cells of a healthy human subject. Lines were stored as stocks in liquid nitrogen. Cells were grown on poly-l-ornithine/ laminin coated 6-well plates (Sigma, St. Louis, MO, USA) and maintained in 70% DMEM, 2% B27, 1% Penicillin/ Streptomycin (Life Technologies Foster City, CA, USA), 30% Ham’s F12 (Mediatech Herndon, VA, USA), with 20 ng/mL bFGF (R&D Systems, Minneapolis, MN, USA), 20 ng/mL EGF, and 5 μg/mL heparin (Sigma). To differentiate cells, we removed growth factors and let cells differentiate for 30 days. Media were changed every 3 days, whether cells were proliferating or differentiating. DNA was extracted from collected cells using QIAGEN’s QIAmp DNA Mini Kit (QIAGEN, cat #: 51304).

### Bisulfite and oxidative bisulfite conversion of human brain genomic DNA

For standard bisulfite conversion, 500 ng of genomic DNA from saliva from each individual was bisulfite converted using the EZ-96 DNA Methylation-Gold kit (Zymo Research, cat. # D5007) and was eluted in 30 μL of water. 1ug of DNA from post-mortem brain tissue was bisulfite converted using QIAGEN’s EpiTect Fast Bisulfite Kit (QIAGEN, Cat. #: 59104), as per manufacturer’s guidelines. Oxidative bisulfite conversion was performed using the CEGX True Methyl kit (Cambridge Epigenetix, Cat. #: CEGXTMS). Briefly, 1ug of DNA from all samples was purified and denatured. DNA from each subject was then split in 2 equal reactions, one of which underwent chemical oxidation followed by bisulfite conversion, while the other underwent mock oxidation (oxidant replaced by water) followed by bisulfite conversion. All bisulfite reactions were cleaned-up using bead-based purification and final elution was in 30ul of elution buffer. Efficiency of oxidation and bisulfite conversion using the CEGX True Methyl kit were evaluated pre- and post-sequencing using spike-in controls, as per manufacturer’s guidelines.

### Targeted-BS sequencing: PCR amplification, library preparation, and customized sequencing

#### Primer design

Bisulfite sequencing primers were designed for each target of interest using the Methyl Primer Express software v1.0 from ThermoFisher Scientific (https://www.thermofisher.com/order/catalog/product/4376041). We designed primers for the first round of PCR without any modifications, adapters, or indices. Optimal amplicon length, especially for oxidative bisulfite sequencing, is 200–300 bp. Amplicon lengths for standard bisulfite conversion for methylation analysis were always less 500 bp to allow for the interrogation of the entire amplicon with high quality scores using 300 bp paired-end sequencing. Although CpGs in the primer sequence were avoided, cytosines in a CpG context in the forward and reverse primers that could not be avoided were replaced with Ys (50% mix of cytosine and thymine) and Rs (50% mix of adenine and guanine), respectively. For the 2^nd^ round PCR primer sets, we designed primers identically to round 1 primers but added universal 22-bp overhangs (CS1 and CS2) on the 5` forward primer and 3`reverse primers, respectively. The CS1 sequence serves as an annealing site for the Read1 customized sequencing primer (LNA-CS1) and CS2 serves as an annealing site for both the Read2 customized sequencing primer (LNA-CS2) and the Index read primer (LNA-CS2rc). When designing primers carrying the CS1 or CS2 sequences, we added three N bases (25% mix of all four bases) between the CS and target sequences to increase base diversity and improve sequencing QC in the early cycle stages. A third pair of PCR primers, for round 3, was designed to add Illumina flow-cell attachment sites (P5 and P7) and a unique 10 base-long non-Illumina single barcode. We used Fluidigm-based barcodes for the first 384 indices (Fluidigm Corporation, California, USA) and our home-made barcodes for beyond 384 indices. These primer sequences targeting specific loci, the CS1, CS2, P5, and P7 adaptor sequences, the indices, and the LNA oligonucleotide sequences can be found in Additional files 1, 2, 3 and 4.

#### Library preparation

**Fig. 1:**
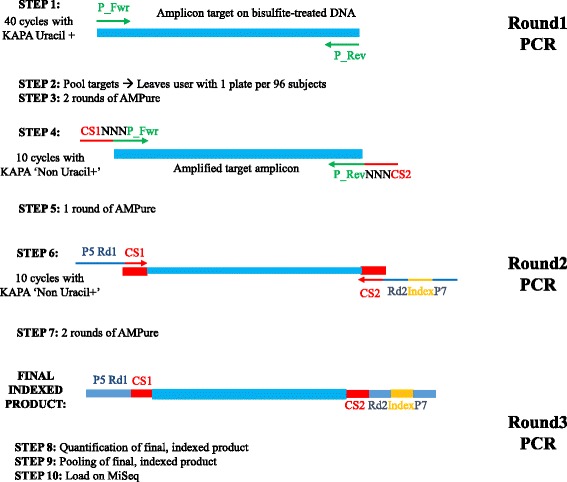
Targeted BS-Seq library preparation requires 3 consecutive PCR reactions. Three successive steps for preparation of targeted BS-Seq libraries include the initial amplification of the loci of interest, the addition of the CS1 and CS2 universal adaptors, and the inclusion of the P5 and P7 adaptors that allow for cluster formation on the flow cell

Figure 1 shows the complete work flow of our library preparation method. An initial PCR reaction was performed on bisulfite or oxidative bisulfite converted DNA using the KAPA HiFi Uracil + ReadyMix (Kapa Biosystems, cat. #: KK2802) and all round 1 bisulfite primer sets, for all targeted regions, in a single reaction per subject. The first PCR for target amplicon generation used the following thermal cycler condition: 95 °C for 3 min, then 38–48cycles of 98 °C for 20s, Tm (60 °C in most cases for multiplexed primer sets) for 15 s, and 72 °C for 15 s, then finished with 72 °C for 30 s. Next, we performed two rounds of purification using Agencourt AMPure XP beads (AMPure) (Beckman Coulter, Cat. #: A63881). We used a 1× AMPure ratio (1:1 AMPure to PCR product) for PCR products for all rounds of PCR. The second round of PCR was performed under the same thermal cycler conditions, but with only 10 cycles. The purpose of the 2^nd^ round of PCR is to add the CS1 and CS2 sequences to each amplicon. While this can, conceivably, be done in Round 1 of PCR, we have much better amplification of targeted regions when the initial PCR includes only primers targeted to the bisulfite converted genome. We also use `non- Uracil + ` KAPA HiFi ReadyMix (Kapa Biosystems, cat. #: KK2602) in PCR Round 2 to reduce costs. The PCR reaction was purified twice with AMPure beads, as performed following Round 1 PCR. Finally, the third PCR reaction with another 10 cycles was performed using the 5` universal primer and 3` barcoding primer sets to add the Illumina flow-cell attachment sequence P5 and P7, as well as single unique barcode. A more detailed description of the universal CS1, CS2, P5, and P7 adaptor additions can be found in Additional file 5. Two double-sided AMpure purifications were performed to complete the library preparation for complete removal of non-purity and primer dimers. Libraries were quantified using the Agilent 2200 TapeStation Instrument and DNA 1000 reagents. Next, we pooled all normalized libraries from all samples into a single reaction tube for sequencing.

#### Custom MiSeq sequencing

To achieve an optimal cluster density of 700–800 K/mm2 for bisulfite-sequencing on the Illumina MiSeq, a final molarity of 2 nm of the pooled samples was used. A 10–20% PhiX spike-in was also included to increase total base diversity, which is essential for obtaining high quality reads during bisulfite sequencing. The PhiX spike-in may need to be further increased if one is sequencing a single region in very large sample sets. Pooled samples were sequenced on the Illumina MiSeq sequencer using the V3 600 cycle kit to perform a 300 bp paired-end sequencing run. For customized MiSeq sequencing, three customized sequencing primers, LNA-CS1 (Read1), LNA-CS2rc (Index Reading), and LNA-CS2 (Read2) primers corresponding to Illumina read1, index, and read2 sequencing primers, respectively, were synthesized in locked nucleic acid form (EXIQON, primer sequences listed in Additional file 2) and loaded onto the corresponding sites on the MiSeq reagent cartridge (see Additional file 6). Illumina Experiment Manager (EM) does not support customized sample running sheet, so an example of a customized sample running sheet in .csv format was created. An example of the .csv sample running sheet is also available in Additional file 7.

### NEXTERA XT library preparation

Libraries were prepared using the NEXTERA XT kit (Illumina, Cat. #: FC-131-1024), as described by Masser et al [7]. Briefly, dual-indexed libraries were generated according to the manufacturer’s protocol. Initial PCRs were performed on bisulfite converted DNA using a ProFlex thermocycler, under the following conditions: 95 °C for 3 min, then 45 cycles of 98 °C for 20s, 58 to 62°c (depending on the primer pair used) for 15 s, and 72 °C for 15 s, and finished with 72 °C for 1 min. Purified PCR products were quantified by Quant-IT Picogreen (Thermo Scientific, cat. #: P7589) and pooled to a final 0.2 ng/μl concentration. A total of 1 ng was used for library generation in a 96-well plate format. Transposome-mediated simultaneous DNA fragmentation and adapter ligation, that is, tagmentation, was performed at 55 °C for 5 min. After the tagmentation reaction, indexing specific PCR primers were added two per well for unique dual indexing. A 12-cycle PCR was performed to amplify the libraries and incorporate the index sequences to the libraries using the following reaction conditions: 72 °C for 3 min and 95 °C for 30s, then 11 cycles of 95 °C for 10s, 55 °C for 30s, and 72 °C for 30s, and finished with 72 °C for 5 min and hold at 10 °C. Amplified libraries were purified using 30 to 50 μl AMPure XP beads (Beckman-Coulter, Brea, CA, USA), and eluted off the beads in 52.5 μl resuspension buffer (provided with Nextera XT). The quality of double-stranded libraries was checked using a High Sensitivity DNA chip run on the Agilent 2100 Bioanalyzer (Agilent Technologies, cat. #: 5067-4626) for size and molarity determination. Based on these metrics, libraries were diluted to 2nM in 10 mM Tris with 0.5% Tween. Equimolar libraries were pooled in equal volumes for denaturation and dilution in HT1 (Illumina, San Diego, CA, USA) buffer. Briefly, 10 μl of pooled NGS library was mixed with 10 μl 0.2 N NaOH for 5 min, then the library was diluted to 20 pM in HT1 buffer. PhiX control libraries were used to increase diversity of base calling during sequencing. 10 nM stock of PhiX library was denatured in 0.2 N NaOH, then diluted to 20 pM in HT1 buffer. Diluted, multiplexed libraries were mixed with diluted PhiX at 4:1 volume ratios, followed by a final dilution to 11pM with HT1 buffer (1 ml final volume). 600 μl was loaded onto the reagent cartridge for MiSeq sequencing. In contrast with the aforementioned Targeted-BS sequencing approach, here classical Illumina sequencing primers were used.

### Reduced Representation Bisulfite Sequencing (RRBS)

RRBS was performed as described previously [8, 9]. Briefly, genomic DNA was extracted from iPSC-NPCs (D28) and digested with MspI. Fragments were end-repaired, Illumina adaptor sequences were ligated, and the libraries were bisulfite converted using Qiagen’s EpiTect Fast 96 Bisulfite Kit. Sequencing was performed on Illumina’s HiSeq 2000 using 50 bp single-end reads. Only CpG sites with coverage greater than 5× were included in the analyses.

### Bioinformatic and statistical analyses

Adaptor sequences were trimmed from the reads when encountered (using Trimmomatic 0.35 with the following parameters: ILLUMINACLIP:Fluidigm_primers.fa:3:30:9 HEADCROP:3 LEADING:20 TRAILING:20 SLIDINGWINDOW:4:20 MINLEN:50, Fluidigm_primers.fa containing the primers). The sequencing reads with a Phred quality score less than 20 were discarded. Bismark v0.14.4 was used to align the remaining reads to the target regions. Bismark was run using Bowtie 2 (version 2.1.0). The extraction of the number of unconverted and converted CpGs for each cytosine was done with the script bismark_methylation_extractor provided with Bismark with the following parameters: -p --no_overlap --cytosine_report --counts -s –bedGraph. The unconverted CpG percentage was calculated for each CpG site as unconverted read counts divided by total read counts. 5hmC percentage at each CpG site was derived as unconverted CpG ratio in bisulfite-only reaction subtracted by the counterpart in oxidation + bisulfite reaction of the same DNA sample.

## Results

### Comparison of targeted BS-sequencing with Nextera XT

We performed target amplification using bisulfite converted DNA (BS-DNA) from post-mortem brain tissue of 16 healthy controls across genomic regions with varying levels of cytosine modifications, as determined from previous genome-wide methylation mapping studies performed in our laboratory. In total, there were three primer sets used in this experiment targeting different regions of the human *OPRK1* gene (see primers sequences in Additional file 3). BS-DNA amplicons were then used for library preparation with either NEXTERA XT or our newly developed targeted BS-Seq approach. In case of NEXTERA XT, samples were pooled with 10–15% PhiX to increase base diversity in the sequencing reaction and analysed on the MiSeq using Illumina sequencing primers and indices. The run, which pooled multiple experiments not described here, produced 11.8 million reads. A total of 1.64 million reads were attributed to the 16 indices (samples) of interest, among which 0.96 million reads passed filtering. 88.26 ± 0.78% of those reads were aligned to the reference human genome. The percent of reads allocated to each of the 16 samples ranged from 3.47 to 10.03%, slightly deviating from the expected 6.25% per sample because of pipetting variability during pooling. Across all samples, the average read number per locus was 66,526 ± 4825, 19,580 ± 1697, and 14,397 ± 1207, which likely reflects the variable efficiency of primer pairs during PCR amplification.

**Fig. 2:**
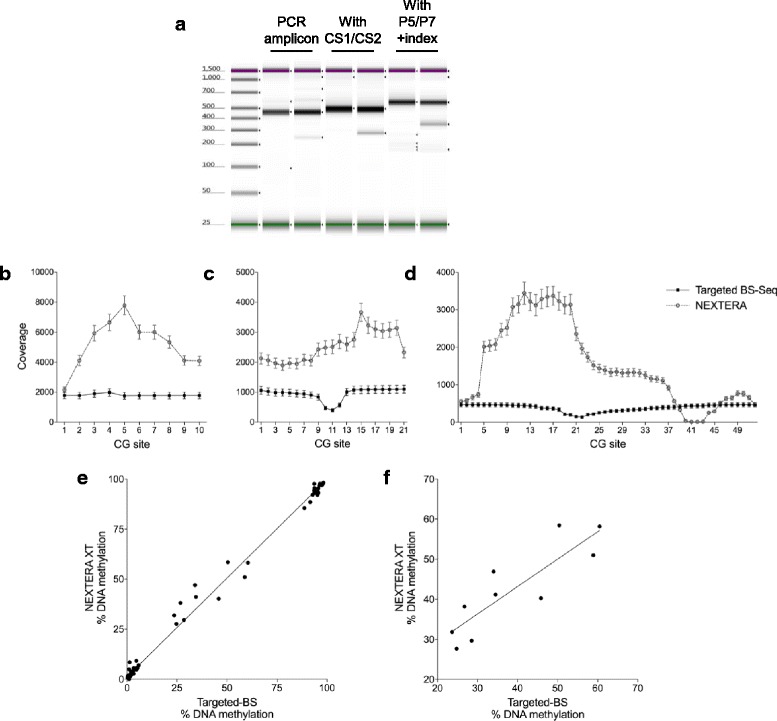
Targeted BS-Seq performs to the same standard as Illumina’s NEXTERA XT kit. Three genomic regions located in the human OPRK1 gene were amplified by quantitative PCR and then used for library preparation with either (i) the commercially available, gold standard NEXTERA XT kit (Illumina) or (ii) our targeted BS-Seq approach. **a** In targeted BS-Seq, successive rounds of PCR are performed to amplify BS-DNA and add universal and reusable primers (CS1 and CS2), followed by adapters to the MiSeq flow cell (P5 and P7 + index). Gel electrophoresis was performed on Agilent’s Tape Station and shows the expected increases in size of PCR products for 2 replicates after each round of PCR. **b–d** Both NEXTERA XT and targeted BS-Seq yielded very high coverages throughout the amplicons of interest, with the exception of 4 CpG sites in NEXTERA XT results (**c, d**). Note that while the difference in coverage between the 2 methods only reflects the amount of material loaded onto the sequencer, the targeted BS-Seq method allows for a more even distribution of coverage along the amplicons and lower variability in coverage across biological samples. Values represent mean ± S.E.M. **e** NEXTERA XT (y-axis) and targeted BS-Seq (x-axis) measured very similar DNA modification levels at all CpGs interogated (*r* ^2^ = 0.9947, *p* < 0.0001), confirming that our methodology matches a gold standard, commercially available solution to call DNA modifications with high precision. **f** This correlation remains highly significant (*r* ^2^ = 0.7476, *p* = 0.0012) even when looking at the 10 CG sites that showed intermediate methylation levels, corresponding to sites with more variable methylation states across individual cells

In case of the targeted BS-Seq approach, we confirmed the quality of library preparation by showing the size shifts indicating the successive inclusions of CS1/CS2 sequences (PCR Round 2) and Illumina’s P5 and P7 flow-cell attachment sequences (PCR Round 3) (Fig. 2a). The pooled samples contained a 10–15% PhiX spike-in, and this run, which pooled additional experiments not described here, produced 182,195 reads that were attributed to the 16 indices (samples) of interest, with 147,010 reads passing filtering. The percentage of reads allocated to each of the 16 samples ranged from 3.49 to 15.94%, with a slight deviation from the expected 6.25% similar to the one described above for NEXTERA. Across all samples, the average read number per locus was 1099 ± 130, 469 ± 62, and 1782 ± 222, reflecting the variability in primer pairs efficiencies during PCR amplification. Considering that each CpG is covered >100×, this does not affect final percent methylation calling.

Among limitations associated with the NEXTERA XT approach is the possibility that coverage within targeted regions may be uneven due to the variable efficiency of transposomes to access and cut DNA regions with different base content, as well as decreasing coverage at the extremities of targeted PCR amplicons [10, 11]. Our data using NEXTERA XT are consistent with these notions, as coverage was significantly lower at both ends of each amplicon (Fig. 2b-d), and a small region covering four CpG sites within one amplicon (Fig. 2d) also showed low coverage. In our targeted BS-Seq approach, since every read for a given amplicon starts and ends at similar positions in the reference genome, we observed much more stable coverage, with only a modest drop at the center of two amplicons (Fig. 2c-d) that, as expected, reflected a drop in Phred quality scores at the extremities of long reads obtained from BS-converted DNA.

**Fig. 3:**
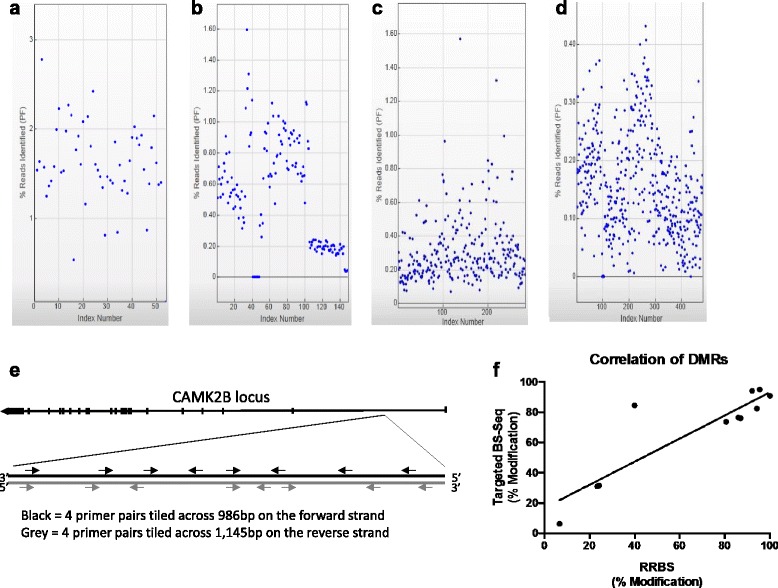
Multiplexed single-barcode system for targeted BS-Seq can accommodate a variety of strategies and can be extended to 478 individual indices. Total reads per index are outputed following each MiSeq run. **a** Using a smaller sample size, we are able to generate data where the percentage of reads allocated to each index is consistent and abundant. **b** Pooling indices with different ratios allows users to control the percentage of reads allocated to individual indices. **c-d** Our targeted BS-Seq protocol and the MiSeq sequencer can accommodate an extended number of indices, beyond 300 (**c**) and 478 (**d**), while still maintaining coverage greater than our threshold of 100×. **a-d** Y-axes correspond to the % of reads identified per indexed subject after sequencing. X-axes correspond to the number of indexed samples loaded onto the MiSeq. **e** Representative diagram of tiled primers on positive and negative strands of 11 loci interrogated by targeted BS-Seq, shown here for the CAMK2B locus as an illustration. These 11 loci were located on the following 7 genes: CAMK2B (1 locus), PAX6 (4), NDRG2 (2), LHX1 (1), ATP1A3 (1), TBR1 (1), and OTP (1). **f** Correlation of % modification values obtained using RRBS or our targeted BS-Seq approach (*r* ^2^ = 0.7922, *p* = 0.0002). The % of modification correspond to the mean values obtained across three biological replicates for both RRBS and targeted BS-Seq

Of the 83 CpGs targeted by the three primer sets at these loci, including the four CpGs with lower coverage, cytosine modification values ranged from 0 to 100% (Fig. 2e), and methylation levels were detected almost identically between our approach and Nextera (*r* ^2^ = 0.9947, *p* < 0.0001). Furthermore, this correlation remains strong when looking specifically at the 10 more variable sites (*r* ^2^ = 0.7476, *p* = 0.0012) (Fig. 2f). We note also that these strikingly similar results were obtained while very distinct coverages were observed across the two methods, which simply reflect the different concentrations of pooled samples loaded onto the MiSeq cartridge. In addition to this comparison with NEXTERA, and as an additional cross-validation of our methodology with alternative technologies, we present, in Fig. 3 (see below), results from the comparison of DNA methylation measures obtained using RRBS and targeted BS-Seq.

Altogether, results strongly suggest that high precision measures of cytosine modifications can be achieved in much higher sample numbers, even in the face of decreasing coverage, as discussed below. Overall, these results confirm the efficacy of our targeted BS-Seq approach to analyze a range of modification levels, without the associated costs and experimental difficulties of traditional, enzyme-based library preparations.

### Medium throughput capabilities of this targeted approach

We next determined the multiplexing capacity of this targeted approach. To do so, we analyzed cytosine modification levels in progressively larger sample sizes with varying number of loci being targeted. First, we used fewer samples to allow for increased coverage per loci in each sample. This run interrogated 26 loci in post-mortem human brain from 54 subjects and included a 15% PhiX spike-in. In total, 16.05 million reads passed a Phred QC score > 30, representing 78.0% of the total reads. We are able to show equal distribution of coverage across samples, with 1–2% of all reads being allotted to each index (Fig. 3a). In a second test, we demonstrate the ability of this method to use different pooling ratios for two different projects, as well as sequencing controls, negative controls, and a 20% PhiX spike-in (Fig. 3b). The first project contained amplicons for seven genomic loci across 96 samples, which required four times higher sequencing depth than the second project that investigated three loci across 40 samples. Four sequencing controls for the second project were also included in this run, and were included at a 4× dilution relative to the samples of the second project. In total, 14.05 million reads passed a Phred QC score > 30, representing 78.7% of the total reads. These data confirm the ability of this methodology to accurately sequence libraries from multiple projects during the same MiSeq sequencing run, thereby adding to the cost savings.

To further test the utility of this methodology in large cohorts, we attempted to pool 288 and then 478 individual indices in a single run. Both runs interrogated four genomic loci in human saliva samples, the first with a 10% PhiX spike-in and the second with 20% PhiX. Although the output per sample is lower, as expected, the crowded flow-cells still generated greater than 85.9 and 74.1% of reads that passed a Phred QC filtering score > 30, respectively, and a minimum coverage of 100× per loci (Fig. 3c and d). When using larger cohorts, equimolar pooling is more challenging because accurate quantification of each sample can be costly. Nevertheless, the minimum coverage was still obtained, even with the larger variance in the percent of reads per sample seen in Fig. 3d. This experiment highlights the ability of this approach to multiplex upwards of 478 indices and many primer sets in a single reaction, targeting all genomic regions of interest at once.

In an attempt to decrease bench time, we also wanted to determine the extent to which primers targeting different loci can be multiplexed during the first round of PCR. In this run, we designed ~16 primers (8 on each of the positive and negative strands), both with and without the CS1 and CS2 sequences included, across 14 loci in 12 samples (Fig. 3e). All 236 primers, corresponding to multiple potential amplicons for each locus, were multiplexed in a single PCR reaction, per sample, to amplify bisulfite converted DNA from iPSC-NPC lines, which was followed by the second and third rounds of PCR to include the CS1/CS2 and P5/P7 sequencing adaptors, respectively. The sequencing run generated 21.7 million reads that passed a Phred QC filtering score > 30 (82.7% of total reads), of which 8.3 million were specific to this project. Each of the 12 indices received between 2.0 and 4.7% of reads that passed filtering (mean = 3.20 ± 0.24%), which is quite close to the expected 3.45% per sample). Impressively, 12 of the 14 loci were successfully amplified and sequenced, which included data from 702 CpGs across 10 kb of DNA. Coverage ranged from 200 to 20,000×, depending on the locus, with a mean of 6771 ± 2132× (Table 1). This range is likely due to the different primer efficiencies in the multiplexed reaction. Although this should be taken into consideration when designing an experiment, having reached a minimum of 100× coverage across all loci confirms the efficacy of this methodology even in such a high multiplexing scenario.

**Table 1:** Quantitative output from highly multiplexed targeted BS MiSeq run

Chromosome	Covered region	Length of the covered region (bp)	Number of CpG sites detected in covered region	Coverage full data set	Number of primer pairs
Chr4	46,391,588–46,392,165	578	44	5407	10
Chr3	11,034,244–11,035,166	923	90	19,607	7
Chr7	44,349,021–44,349,856	836	62	21,495	8
Chr11	31,820,209–31,820,665	457	26	1799	8
Chr11	31,825,606–31,827,393	1788	110	8232	16
Chr14	21,542,358–21,543,245	888	29	194	8
Chr14	21,558,269–21,559,093	825	49	3117	7
Chr17	35,299,547–35,300,293	747	61	1365	10
Chr19	42,489,794–42,490,445	652	16	5089	7
Chr20	61,992,854–61,993,598	745	53	1830	7
Chr2	162,284,277–162,284,993	717	58	369	7
Chr5	76,925,266–76,926,368	1103	104	12,744	8

Finally, for three of the samples interrogated during the latter highly multiplexed run, DNA methylation measurements were already available from previous RRBS experiments (conducted by our group in the context of studies investigating DNA methylation changes during iPSC-NPC differentiation). Results show that a significant correlation was observed between the % modification from RRBS and % modification from our targeted sequencing approach across these loci (*r* ^2^ = 0.7922, *p* = 0.0002, see Fig. 3f), demonstrating the comparability of our approach to a restriction enzyme-based library preparation methodology.

### Target validation using oxidative bisulfite sequencing (oxBS)

**Fig. 4:**
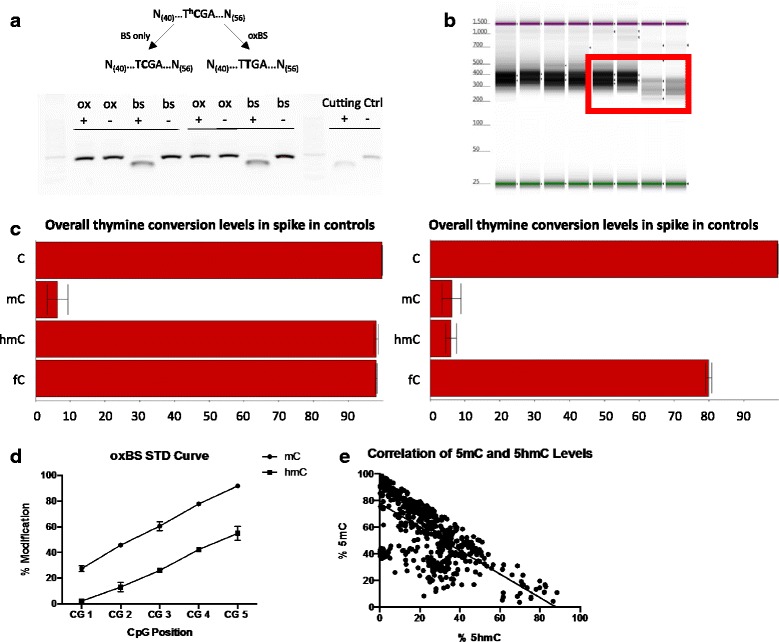
Targeted oxBS accurately differentiates 5mC from 5hmC in post-mortem brain. **a** A 100 bp spike-in digestion control sequence contains a 5hmC mark at the TCGA Taq α1 restriction enzyme digestion site located at position 42 (^**h**^ **C**). An efficient oxidation reaction will convert this 5hmC to thymine after bisulfite conversion, which will prevent the enzyme from cutting (**T** in the oxBS arm). In the bisulfite-alone reaction, the 5hmC will remain a cytosine after the bisulfite reaction and will be cut by the enzyme (**C** in the BS arm). As per manufacturer’s guidelines, digestion control reactions were run on a 1.5% agarose gel with (+) and without (-) the restriction enzyme and an additional cutting control was included, also with (+) and without (-) the restriction enzyme. **b** The targeted bisulfite sequencing approach presented in this manuscript involves consecutive PCR reactions to add the universal adaptors and indices, therefore, we expected to see an increase the size of the amplicons. Gel electrophoresis was performed on Agilent’s Tape Station. Each lane represents a different subject. In the *red box*, the 3^rd^ and 4^th^ bands represent amplicons with the universal adaptors, while the 1^st^ and 2^nd^ bands include the universal adaptors and the indices. **c** During the target amplification, primers were also designed to interrogate spiked-in sequencing controls that contain a variety of 5hmC, 5mC, 5fC, and C bases (provided by manufacturer). Conversion rates of cytosine to thymine in the oxBS (*left*) and BS (*right*) reactions were as expected, with 5hmC being converted to thymine after oxidation and bisulfite conversion. **d** Representative CpGs showing varying levels of 5mC and 5hmC between 0 and 100% were selected to design a standard curve showing the range of detection of 5hmC and 5mC in the loci we interrogated. **e** The correlation of %5mC and %5hmC across all 30 CpGs shows an inverse relationship between the two modifications

Recent studies have uncovered 5hmC as a new, stable epigenetic mark [12–14] that, in contrast with the negative effect classically associated with 5mc, positively affects gene expression, at least in brain tissue [15–17] and immune cells [18]. Because bisulfite treatment can convert both 5hmc and 5mc [19], we assessed whether our targeted BS-Seq method could differentiate 5mC from 5hmC. To distinguish 5hmc and 5mc, a chemical or enzymatic oxidation prior to bisulfite conversion is required when analysing DNA [20, 21]. To determine if our approach can also be used to evaluate 5hmc, we performed oxidative (ox) BS, a specific chemical oxidation of 5hmC to 5fC, prior to bisulfite treatment. The purpose of this oxidation step is to generate 5fC, which is no longer resistant to bisulfite-mediated C-T conversion [22]. First, we confirmed the efficiency of the oxidation and bisulfite reactions by digesting specialized spike-in sequences with known 5hmC at an enzymatic cut site. The TCGA Taq α1 cut site in these spike-in sequences contain a 5hmC at the cytosine position. The combination of the oxidation reaction and the bisulfite conversion will convert 5hmC to thymine and prevent the enzyme from cutting, while the bisulfite reaction alone will maintain this cut site (Fig. 4a). Results from this experiment demonstrate that our technical procedures work as anticipated with a known standard. To test this procedure on a real example, we used DNA from post-mortem brain tissue of 19 psychiatrically healthy individuals, targeted seven genomic loci for 5hmC and 5mC mapping, and proceeded with the medium throughput protocol (primer sequences can be found in Additional file 4). Following library preparation, we confirmed the addition of the universal primer sequences and indices, as illustrated by the expected increase in molecular weights of our amplicons (Fig. 4b). Following sequencing, we first assessed the cytosine to thymine conversion rates to confirm the efficacies of the oxidation and bisulfite reactions we observed prior to sequencing. In the oxidation reaction, we observe a near complete conversion of 5hmC bases to thymine, while this is abolished in the bisulfite-alone reaction (Fig. 4c).

The sequencing run, which included 94 indices from two different experiments, produced 26.7 million reads passing a Phred QC score greater than 30. Of these, 11.5 million reads were allotted to the 38 indices (mean reads per index = 301,350 ± 5883), which corresponded to the oxBS and BS fractions from the 19 samples used in this manuscript, and covered 30 CpGs across the seven targeted genomic regions of interest. Using a representative group of CpGs, chosen based on having varying levels of 5mC and 5hmC between 0 and 100%, we detect a range of 20–100% 5mC and 0–60% 5hmC levels with little variation across subjects (Fig. 4d), suggesting good internal consistency across samples. Furthermore, we assessed the distribution of both 5mC and 5hmC levels across the all the loci we investigated and show an inverse relationship between 5mC and 5hmC (Fig. 4e), which could be expected given their relationship to one another. These data confirm the extended use of our targeted validation approach to effectively analyse 5hmC following chemical oxidation.

## Discussion and Conclusions

The goal of the present study was to implement a medium throughput methodology to interrogate DNA methylation and hydroxymethylation in multiple biological samples and genomic regions in a cost-effective and timely way. First, we compared the proposed targeted BS-Seq methodology with a commercially available gold-standard, in this case the NEXTERA XT kit, and showed that both methods yield high-coverage data and very similar levels of DNA cytosine modifications. We, then, extended the utility of this methodology to simultaneously sequence up to 478 individual indices in a single MiSeq run. Finally, we confirmed the ability of this targeted BS-Seq method to be combined with oxBS-Seq to differentiate 5mC from 5hmC in genomic DNA.

In this study, we used the NEXTERA XT approach as a point of comparison to determine the efficacy of our methodology. NEXTERA XT is an excellent and widely used library preparation kit because it allows for the processing of large amplicons, up to several kb according to manufacturer. However, not only does the bisulfite conversion step lead to fragmentation of the input DNA template, making it difficult to PCR amplify fragments larger than 500 bp, but incomplete cytosine to thymine conversion rates are higher for longer amplicons [23]. As such, the ability to maximize the potential of library preparation technologies for cytosine modification analyses, which could potentially accommodate longer DNA amplicons (such as Nextera XT), is greatly reduced. Similarly, although Illumina’s V3 MiSeq kit allows for 600 cycles, the current technology generates the best quality reads for first 200–250 bases. For these reasons, the optimal bisulfite-PCR amplicon length should be restricted to a maximum of 500 bp. For targeted regions greater than 500 bp, creating tiled primer pairs allows for the interrogation of the entire region of interest. Therefore, while the NEXTERA XT kit is a feasible solution, we have produced data of similar quality at a much lower price using a targeted BS-Seq approach.

The strength of this method is the high level of mutiplexing built into the primer design and the ability to index hundreds of samples on a single reaction of a benchtop sequencer. Currently, targeted cytosine modification mapping requires time-consuming primer design and primer assessment to ensure accurate amplification. We have circumvented this problem by using many primer sets for a given locus combined with parallel sequencing of amplicons. While more primer sets virtually guarantee off-target effects, they also act in unexpected ways at the target region to maximize the coverage. We have demonstrated, in this study, that this multiplexing can also work across multiple regions in the same reaction tube. While this leads to more off-target effects, we show that average coverage is still above the 100× threshold for regions of interest, and the downstream bioinformatic processing remains the same since non-target regions can be discarded. Furthermore, the simultaneous amplification of multiple targets using multiplexed primer sets in a single PCR reaction can save input BS-DNA. This is especially important in certain clinical or experimental cases where input DNA is scarce.

The increased customized index capacity for multiple target amplicons and, importantly, the possibility to differentiate 5mC and 5hmC provides strength to the validity and usefulness of our methodology, and shows several clear advantages over other commercially-available library preparation and indexing systems. In particular, this method does not require expensive methylated primers or oligonucleotides. Furthermore, while we show, in the present manuscript, the feasibility of interrogating up to 478 samples using our 10 nucleotide single barcoding system, the maximal capacity could be mathematically expanded to 1536 indexes. To achieve this same level of multiplexing using commercial kits, one would need to buy multiple sets of each indexing system, which drastically increases the associated costs. Although increasing the number of indices in a single run will inherently decrease the coverage per loci, this minor limitation can be easily overcome by running a second MiSeq reaction, which is still less expensive than using other library preparation kits.

Current methodologies for bisulfite sequencing are performed on the whole genome, such as WGBS, or on a representative fraction, such as RRBS. However, the accessibility of the sequencing technologies remains limited because they require exhaustive sequencing to achieve sufficient coverage. As such, exploratory experiments are being conducted on small sample sizes, and often yield results based on coverage less than 10×. Here, we present a methodology that will allow researchers to validate WGBS or RRBS findings in an extended sample size at a reasonable cost, while ensuring sufficient coverage.

This technology is not only suitable as a validation tool, but it can also serve for additional needs, from candidate gene studies to clinical diagnostic procedures. In candidate genes studies, numerous genomic regions can be analyzed simultaneously by pooling multiple PCR products across large sample sizes. In clinical settings, high-resolution data about cytosine modifications, including non-CpG methylation, can be collected while reducing the cost of NGS approaches. In its entirety, the combination of our BS-Seq library preparation method with next-generation sequencing on Illumina’s MiSeq benchtop sequencer represents the ideal platform for studying cytosine modifications, regardless of phenotype or field of study.

## Additional files

Additional file 1: Sequences of universal primers and indices used to achieve the multiplexing of up to 478 samples. (XLSX 9 kb)
Additional file 2: LNA oligonucleotides used for customized sequencing on the MiSeq platform. (XLSX 8 kb)
Additional file 3: Primers used for amplification of genomic DNA using NEXTERA XT and targeted BS-Seq (Fig. 3). (XLSX 9 kb)
Additional file 4: Primers used for amplification of genomic DNA in oxBS experiments (Fig. 4). (XLSX 10 kb)
Additional file 5: Further to the information provided in Fig. 1, Additional **Figure S1** provides additional details regarding the CS1, CS2, P5, and P7 primer sequences, as well as the location of the barcode. (PPTX 144 kb)
Additional file 6: Using this customized BS-Seq method requires users to modify the sequencing reagent cartridge provided by Illumina. These are the operational steps for performing customized MiSeq sequencing run with customized sequencing primers (CS1, CS2, and CS2rc). Arrows indicate the transferring of fluid from fro one tube number to another on Illumina’s reagent cartridge. (1) The contents of tube 12 should be placed in tube 18. (2) The contents of tube 13 should be placed in tube 19. (3) The contents of tube 14 should be placed in tube 20. (PPTX 1142 kb)
Additional file 7: csv sample running sheet. (CSV 19 kb)

## Abbreviations

5hmC: 5-hydroxymethylcytosine
5mC: 5-methylcytosine
AMPure: Agencourt AMPure XP beads
BS: Bisulfite
DMRs: Differentially methylated regions
iPSC-NPC: Induced pluripotent stem cell-derived neural progenitor cells
NGS: Next-generation sequencing
oxBS: Oxidative bisulfite
RRBS: Reduced representation bisulfite sequencing
WGBS: Whole-genome bisulfite sequencing
