# Supplementary figures and images for: Medium throughput bisulfite sequencing for accurate detection of 5-methylcytosine and 5-hydroxymethylcytosine
Source: BMC Genomics. 2017 Jan 18;18:96. doi: 10.1186/s12864-017-3489-9 (PMC5242011; doi:10.1186/s12864-017-3489-9)

## Slide 1
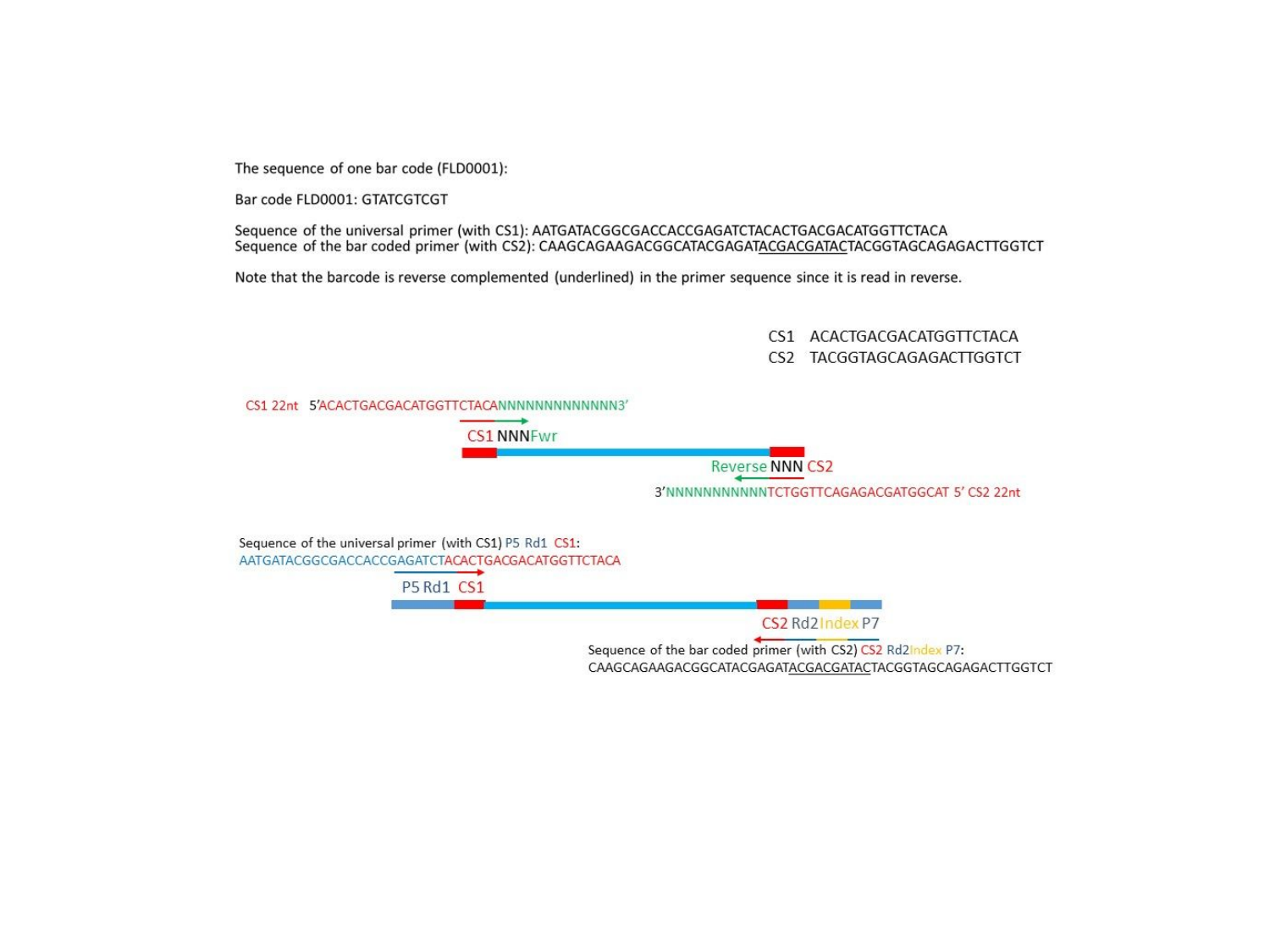

Supplement: Additional file 5: — Further to the information provided in Fig. 1, Additional Figure S1 provides additional details regarding the CS1, CS2, P5, and P7 primer sequences, as well as the location of the barcode. (PPTX 144 kb) [file 12864_2017_3489_MOESM5_ESM.pptx]

## Slide 1
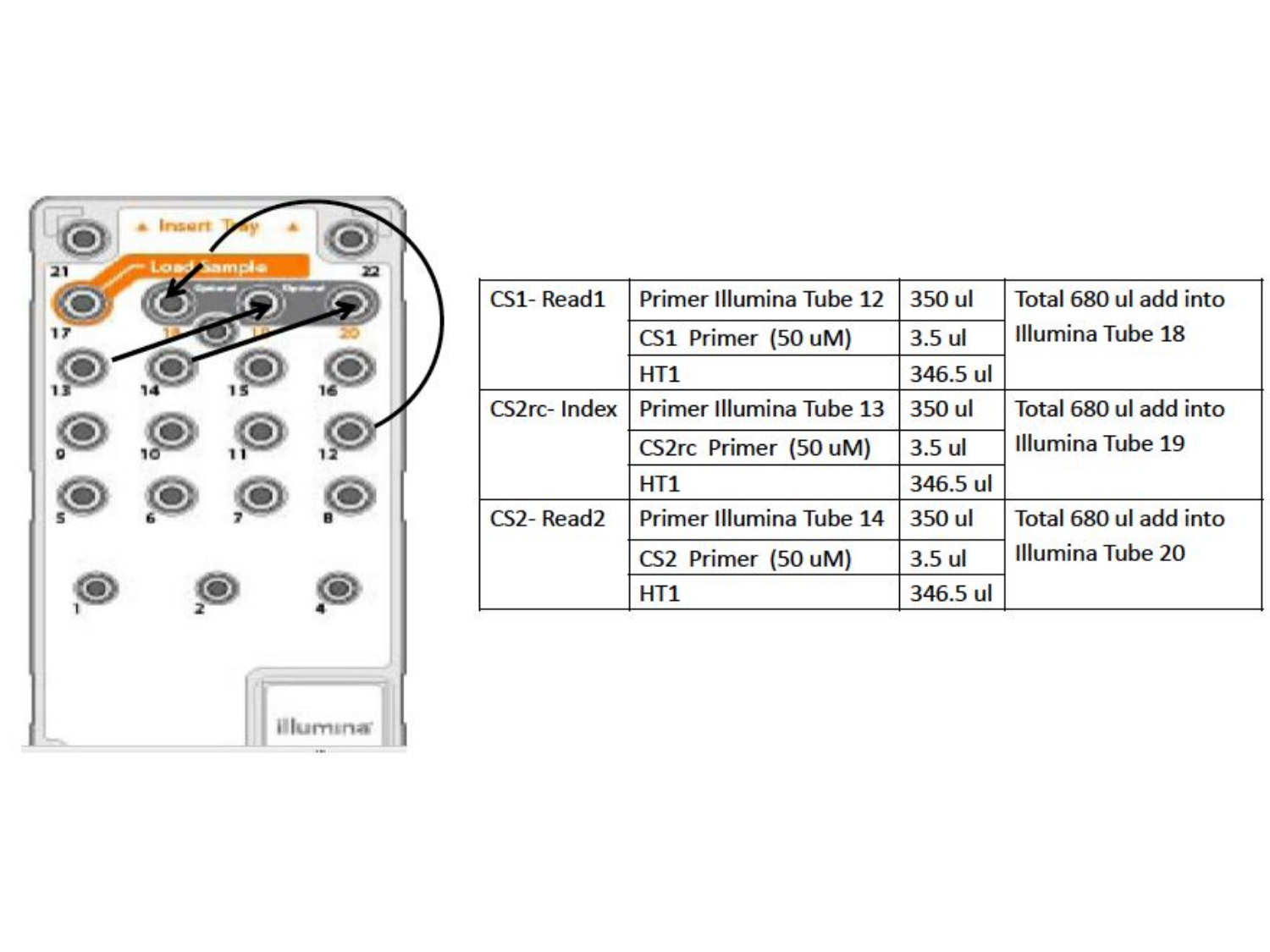

Supplement: Additional file 6: — Using this customized BS-Seq method requires users to modify the sequencing reagent cartridge provided by Illumina. These are the operational steps for performing customized MiSeq sequencing run with customized sequencing primers (CS1, CS2, and CS2rc). Arrows indicate the transferring of fluid from fro one tube number to another on Illumina’s reagent cartridge. (1) The contents of tube 12 should be placed in tube 18. (2) The contents of tube 13 should be placed in tube 19. (3) The contents of tube 14 should be placed in tube 20. (PPTX 1142 kb) [file 12864_2017_3489_MOESM6_ESM.pptx]
